# Artificial intelligence and teacher competence: a scoping review of assessment, analytics, and professional development

**DOI:** 10.3389/frai.2026.1901449

**Published:** 2026-07-27

**Authors:** Nurseit Baizhanov, Batyr Sharimbayev, Zhairan Churbanova, Akzhan Khaidarova, Lyazzat Shinetova

**Affiliations:** 1 National Testing Center, Astana, Kazakhstan; 2 Department of Information Systems, SDU University, Kaskelen, Kazakhstan

**Keywords:** artificial intelligence, learning analytics, machine learning, teacher competency, teacher professional development

## Abstract

This scoping review maps how artificial intelligence (AI) is being connected to teacher competence in recent research. The review was based on 33 peer-reviewed articles published in 2022–2026 and identified through a bounded Web of Science search. Its purpose was not to evaluate intervention effectiveness, but to describe the extent, range, and nature of the available evidence on AI, machine learning (ML), and learning analytics (LA) in teacher assessment, modeling, and professional development within this indexed corpus. The mapped literature suggests two broad lines of work. One uses AI, ML, LA, and computational psychometrics to assess teaching practice, model teacher development, or measure AI-TPACK-related competence. The other treats AI as part of what teachers themselves need to know and do. Instruments represented in the corpus, such as TAICS, T-GAIC, AI-SRLS, AI-TPACK, and RAIS, broaden the concept of competence to include AI literacy, self-efficacy, ethical reasoning, readiness, and teacher–AI co-teaching. The review found frequent use of supervised machine learning, regularized regression, EFA, CFA, SEM, and learning analytics, but limited reported use of explainable AI, subgroup fairness analysis, multimodal validation, and longitudinal designs. Quality appraisal indicated stronger support for measurement-structure claims than for causal claims about professional-development effectiveness or high-stakes AI deployment.

## 1 Introduction

AI technologies have moved from a peripheral topic in education to a practical element of instructional design, assessment, and professional learning. This shift changes the meaning of teacher competence: teachers are increasingly expected to interpret AI-generated outputs, design learning activities around AI-supported tools, and make informed ethical decisions about their use (Celik et al., 2022; Li et al., 2025).

Two broad research directions frame this review. The first uses artificial intelligence to assess or model teachers, drawing on ML, LA, and related computational techniques to analyse teacher portfolios, classroom observations, accreditation records, or survey data (Rashid, 2023; Zhong, 2023). The second studies AI as part of teachers' own professional competence, especially as generative AI requires new forms of pedagogical, technical, and ethical judgement (Shi, 2025; Tan et al., 2025b).

Three contextual studies outside the scoping review's core corpus help position the present review. Almubarak et al. (2025) proposed a deep-learning framework for assessing teacher performance from classroom interaction data and reported that automated modeling can capture instructional interaction patterns relevant to teacher evaluation. Wu et al. (2026) examined AI-related technostress among middle school teachers and showed that AI adoption also creates psychological and organizational pressures that must be considered when discussing teacher competence. Tammets and Ley (2023) developed a conceptual model and illustrative case for integrating AI tools into teacher professional learning, emphasizing that AI-supported professional development requires pedagogical design rather than tool adoption alone.

The period from 2022 to 2026 is important because large language models made AI tools more visible in everyday teaching practice. During the same period, AI-TPACK and related competence frameworks became more established, while teacher-facing ML and LA research began to expand beyond earlier student-focused analytics (Cao et al., 2026; Zambrano-Romero et al., 2025).

These developments raise several conceptual and methodological problems. Competence models now include constructs such as generative AI competence, AI self-efficacy, AI readiness, self-regulated AI learning, and AI-TPACK, but these constructs are not always compared systematically. At the same time, ML- and LA-based evaluations of teaching quality often use complex models without sufficient attention to explainability, fairness, and generalizability (Zhong, 2023; Chiu et al., 2025). Research on AI-supported teacher professional development shows a related issue: pedagogical innovation is advancing faster than evidence on validity, transferability, and long-term impact (Dogan et al., 2025; Salas-Pilco et al., 2022).

To address this conceptual fragmentation, the review uses the distinction between *AI about teachers* and *AI with teachers*. The first term refers to systems that make teachers visible as objects of analysis, prediction, or evaluation. The second refers to systems in which teachers use, interpret, adapt, or co-design AI-supported practices. This distinction is not intended to replace existing competence models such as AI-TPACK, TAICS, or T-GAIC; rather, it adds a relational layer that clarifies whether AI is positioned as an external analytic observer of teachers or as a partner in teachers' professional action (Shi, 2025; An et al., 2026; Chiu et al., 2025).

This review addresses that synthesis gap by mapping 33 peer-reviewed studies retrieved from the Web of Science database from 2022 to 2026. The aim is to scope how AI, ML, and LA are being used to assess, model, or support teacher competence; which competence domains and methods are visible in the literature; and where the main gaps in this bounded evidence base lie.

The review is framed by three scoping questions:

**RQ1**. What is the extent and range of peer-reviewed evidence on the use of AI, ML, and LA in teacher-competence research from 2022 to 2026?

**RQ2**. What methods, data sources, competence constructs, and professional-learning contexts are represented in this evidence base?

**RQ3**. What research gaps and future directions are visible, particularly in relation to validity, explainability, fairness, longitudinal designs, and regional coverage?

Five synthesis outputs arise from this review: (i) the *AI about teachers*/*AI with teachers* framework, which clarifies whether AI is positioned as an external analytic observer or as a partner in teachers' professional action; (ii) a competence synthesis map that brings together the growing number of competence tools; (iii) a method-role classification for AI, ML, and LA; (iv) competence mapping that links competence strata to dominant operationalizations; and (v) a research-gap and professional-development roadmap.

## 2 Materials and methods

### 2.1 Scoping review design

This study was designed as a scoping review rather than as an effectiveness review. The purpose was to map the extent, range, and nature of research activity on AI and teacher competence, not to estimate pooled effects, rank interventions, or determine whether particular AI-supported professional-development models work better than others. The review followed the scoping-study framework proposed by (Arksey and O'Malley 2005) and the refinements suggested by Levac et al. (2010); operational decisions about eligibility, charting, and synthesis were also aligned with updated guidance for conducting scoping reviews (Peters et al., 2020). In practical terms, the review proceeded through five linked stages: identifying the review questions; identifying potentially relevant studies; selecting studies for inclusion; charting the data; and collating, summarizing, and reporting the results. Reporting was guided by the PRISMA extension for scoping reviews (PRISMA-ScR) (Tricco et al., 2018). No review protocol was prospectively registered in OSF, PROSPERO, or any other public registry. This is acknowledged as a limitation of the review process. Although, no public protocol registration was completed, the review was developed with input from experts at the National Testing Center (NTC), who contributed domain expertise on teacher competence, assessment practice, and the interpretation of the findings.

### 2.2 Identifying the review questions

The questions were framed in scoping-review terms. They focus on what evidence exists, how that evidence is distributed across methods and competence constructs, and what gaps can be seen in the mapped literature. They do not ask whether AI-based assessment, analytics, or professional development is effective in a causal sense. This framing is important because the literature includes psychometric validation studies, machine-learning studies, learning-analytics studies, conceptual papers, and professional-development interventions that are not directly comparable through meta-analysis.

### 2.3 Search strategy and information source

The search was deliberately bounded. The Web of Science Core Collection was used as the information source because it provides broad coverage of peer-reviewed education, educational technology, computer science, and interdisciplinary research. This choice made the review manageable and reproducible, but it also means that the results should be read as a map of a defined indexed corpus rather than as an exhaustive account of all work on AI and teacher competence. No additional bibliographic databases, gray-literature sources, dissertation databases, or preprint servers were searched. The last search was run on 2 May 2026. The search period covered records with publication years 2022–2026 that were indexed in Web of Science up to 2 May 2026. The full database syntax, field tag, filters, and limits were as follows:


  Database: Web of Science Core Collection
  Search field: Topic (TS; title, abstract,
  author keywords,
                Keywords Plus)
  Search date: 2 May 2026
  Search period: publication years 2022-2026,
  through the search date
  
  TS=((‘‘teacher competence*'' OR ‘‘teacher professional
  competence*'')
      AND (‘‘artificial intelligence'' OR
      ‘‘machine learning''
      OR ‘‘learning analytics''))
  
  Limits: document type = Article OR Review Article;
          language = English;
          publication years = 2022 OR 2023 OR 2024 OR 2025
          OR 2026


The publication-year limit was chosen because it captures the recent growth of teacher-facing AI research after the rapid public adoption of generative AI tools, while still including early work on ML and LA approaches to teacher assessment. Because the search was intentionally limited to one database and a focused set of competence terms, the review does not claim exhaustive coverage of all related literature.

### 2.4 Eligibility criteria and study selection

Studies were included if they focused on teachers, including in-service or preservice teachers in K-12, higher education, or vocational settings, and if they used AI, ML, or LA to assess, model, predict, describe, or support teacher competence. Studies were also eligible when AI itself was treated as an object of teacher competence, for example through AI literacy, AI-TPACK, generative-AI competence, readiness, self-efficacy, or ethical use. Studies were excluded if they focused exclusively on students, used AI only as an instructional tool without any teacher-competence component, or were non-empirical opinion pieces.

**Figure 1:**
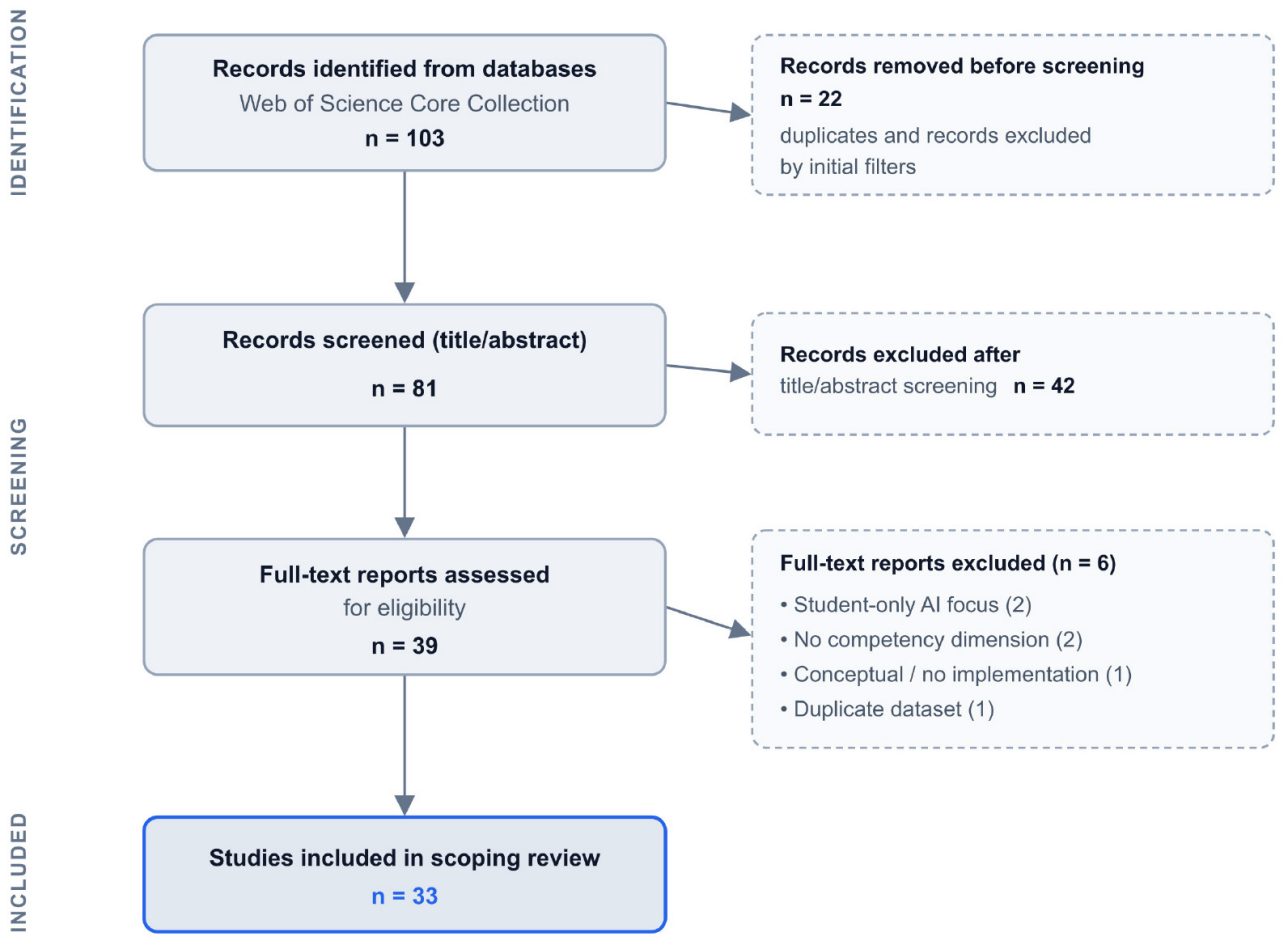
Flow of article screening and study selection.

The same criteria were applied during screening. Screening was conducted in two stages by four reviewers (B.S., Z.C., A.K., and L.S.). At the title-and-abstract stage, each record was screened independently by two reviewers. At the full-text stage, each potentially eligible article was again assessed independently by two reviewers against the eligibility criteria. Disagreements were first discussed by the reviewer pair; unresolved cases were adjudicated by N.B. before final inclusion. No screening reliability statistic, including percent agreement or Cohen's κ, was recorded prospectively; this absence is reported as a limitation of the review record. Title and abstract screening reduced the total of 81 records to 39 potentially relevant studies. Full-text screening then produced the final corpus of 33 studies. Figure 1 presents the study selection flow. Reporting followed the PRISMA-ScR guidelines; the completed PRISMA-ScR checklist is provided as Supplementary Table S3 (Tricco et al., 2018). The cleaned screening-decision log is provided as supplementary review data. In line with PRISMA-ScR, the flow diagram is used to make the boundaries of the mapped corpus transparent rather than to imply that the review exhausts every possible database, keyword variant, or gray-literature source.

### 2.5 Data charting and analytical approach

A standardized charting matrix was used to capture bibliographic data, study design, participants, education level, country or region, and competence models. It also recorded AI, ML, and LA techniques, educational data sources, validation procedures, performance indicators, principal findings, and reported limitations. Data charting was completed by B.S., Z.C., A.K., and L.S. using a shared extraction form. Each included article was charted by one reviewer and checked by a second reviewer. Disagreements or uncertain coding decisions were resolved through discussion, with N.B. consulted when consensus was not reached. To make the synthesis auditable without overloading the main text, Table 1 provides a condensed five-column coding table for all 33 included studies. The full extraction matrix, including country or region, sample, data source, main findings, limitations, and quality notes, is provided as Supplementary Table S1. Fields not available in the manuscript or source record are marked as not reported rather than inferred. Supplementary Table S2 maps the method-role and competence-stratum categories used in the Results back to the relevant extraction-matrix rows. The screening-decision file records title-and-abstract and full-text screening decisions, with full-text exclusion reasons where applicable.

**Table 1:** Condensed coding table for the 33 included studies.

Study	Design/source	AI/ML/LA method	Competence focus	Synthesis role/quality
Celik et al. (2022)	Systematic review; Published AI-for-teachers literature	Systematic review/narrative synthesis	Promises, challenges, and teacher-facing AI competence	Evidence base; assessment and AI-with-teachers context; Tier 3, moderate
Tan et al. (2025a)	Systematic review; AI in teaching and teacher PD literature	Systematic review	AI in teaching and teacher professional development	Evidence base; PD and AI-with-teachers context; Tier 3, moderate
Li et al. (2025)	Systematic review; AI integration in teacher PD literature	Systematic review using adapted technology-based learning model	AI integration in teacher PD; pedagogical AI competence	PD roadmap; pedagogical integration; Tier 3, moderate
Rashid (2023)	ML-based predictive/evaluative study; Accreditation evaluation records	k-means clustering and binary logistic regression	Teacher academic development from accreditation perspective	Predictive-evaluative; AI about teachers; Tier 2, moderate
Zhong (2023)	ML-based teaching-quality evaluation; 16 teaching-quality assessment parameters	GA-SVM compared with SVM	Teaching quality/teacher evaluation	Predictive-evaluative; AI about teachers; Tier 2, moderate-to-lower
Yoon and Kim (2025)	ML explanatory study; TALIS 2018 dataset	LASSO-based machine learning	Teachers' positive perceptions of PD experience	Multilevel/explanatory; AI about teacher development; Tier 2, moderate
Luo and Yang (2022)	Neural-network teaching study; Smart classroom interaction data	CNN and LSTM neural networks	Teacher–student interaction quality/teaching quality	Predictive-evaluative; AI about teachers; Tier 2, lower-to-moderate
Wu (2022)	ML competence-model study; Online teaching environment indicators	Improved machine-learning algorithm	Primary and secondary teachers' competence model	Predictive/evaluative and integration-gap evidence; Tier 2, lower-to-moderate
Cao et al. (2026)	ML-based multilevel study; Teacher-level AI-TPACK and school-level digital-strategy data	Machine learning with multilevel modeling	AI-TPACK; AI ethics; AI-TCK; school digital strategy	Multilevel-explanatory; pedagogical AI competence; Tier 2, moderate-to-higher
Shi (2025)	Psychometric instrument development/validation; Teacher survey responses to generative-AI competence items	EFA/CFA and conventional psychometric validation	T-GAIC; generative-AI competence areas including prompting, output evaluation, and ethics	Psychometric-validation; GenAI competence content; Tier 1, higher-to-moderate
Xiao et al. (2025)	Professional-development feasibility/intervention study; Hybrid-flexible AI competence training data	Empirical feasibility study of hybrid-flexible training	Teachers' AI competence	PD roadmap; institutional barrier evidence; Tier 4, moderate-to-lower
Zhang et al. (2025)	Psychometric scale development/validation; Teacher responses to AI-SRLS items	Psychometric validation grounded in Zimmerman's self-regulation theory	AI self-regulated learning (AI-SRLS)	Psychometric-validation; AI-mediated self-regulated development; Tier 1, higher-to-moderate
An et al. (2026)	Psychometric scale development/validation; AI-TPACK scale responses	AI-TPACK scale development and validation	AI-TPACK for human and AI concurrently as teachers	Psychometric-validation; pedagogical AI and human–AI co-teaching; Tier 1, higher-to-moderate
Chiu et al. (2025)	Psychometric scale development/validation; Teacher responses to TAICS scale	EFA/CFA; measurement invariance noted in manuscript	AI competence self-efficacy; AI knowledge, readiness, ethics, assessment	Psychometric-validation; foundational literacy, ethics, readiness; Tier 1, higher
Delcker et al. (2025)	Instrument development/validation; Teacher self-perception instrument data	Evidence-based instrument development; psychometric assessment	Teachers' self-perceptions of AI competence	Psychometric-validation; self-efficacy/readiness/ethics/self-regulation; Tier 1, moderate-to-higher
Elizondo-Garcia (2026)	Scoping review; Medical education and AI/vibe-coding literature	Scoping review	Vibe-coding/AI-assisted coding as teaching competence	GenAI-specific competence content; Tier 3, moderate-to-lower
Ding et al. (2024)	Professional-development intervention/case-based study; Teacher PD using different case types	Case-based PD design	Teacher AI literacy and AI integration	PD roadmap; foundational AI literacy; Tier 4, moderate
Tan et al. (2025b)	Professional-development intervention study; Intelligent-TPACK PD intervention data	PD intervention based on i-TPACK framework	Teachers' AI competence; i-TPACK; ethics in practice	PD roadmap; pedagogical AI competence; ethics-as-practice; Tier 4, moderate
Ramazanoglu and Akın (2025)	Psychometric scale development/validation; Teacher responses to AI readiness items	Scale development and validation	AI readiness; self-efficacy/readiness	Psychometric-validation; diagnosis phase of PD roadmap; Tier 1, higher-to-moderate
Brandão et al. (2024)	Integrative literature review; Teacher PD and generative-AI literature	Integrative literature review	Generative AI professional development; ethics education	PD roadmap; ethics-as-practice; GenAI specificity; Tier 3, moderate
Tan et al. (2025c)	Quantitative mediation/SEM study; Teachers' AI competence, TPACK, and teaching-performance data	Mediation analysis/SEM	AI competence, TPACK, teaching performance	Pedagogical AI competence; PD roadmap; Tier 1, moderate
Zambrano-Romero et al. (2025)	Systematic review; ML in university teaching-quality literature	Systematic review of ML techniques	Teaching quality of university teachers	Predictive-evaluative method map; limitations/gaps; Tier 3, moderate
Dogan et al. (2025)	Systematic review; AI professional-development literature	Systematic review of TPACK, designs, and effects	AI PD, TPACK, teacher learning	Evidence-base gap; PD roadmap; Tier 3, moderate
Fakhar et al. (2024)	Systematic review/framework paper; Online continuous professional-development literature	Systematic review toward AI-based CPD framework	AI-based online continuous professional development	PD framework; regional balance/gap evidence; Tier 3, moderate-to-lower
Aravantinos et al. (2026)	Systematic review; K-12 AI integration and teacher PD-needs literature	Systematic review	AI literacy and professional-development needs	PD needs; foundational AI literacy; adoption barriers; Tier 3, moderate
Salas-Pilco et al. (2022)	Systematic review; AI and LA in teacher education literature	Systematic review	AI and learning analytics in teacher education	Descriptive-analytic; LA dashboards and analytics; evidence-base gaps; Tier 3, moderate
Lameras and Arnab (2022)	Exploratory review; AI in education literature	Exploratory review	AI affordances for teachers and professional agency	Descriptive-analytic; professional-learning support; AI with teachers; Tier 3, lower-to-moderate
Yau et al. (2025)	Learning-analytics/qualitative-network study; Interview transcripts from teachers undergoing PD	Epistemic Network Analysis (ENA)	Competence to teach AI; TPACK components	Descriptive-analytic; competence-pattern mapping; Tier 2, moderate
Ahmad et al. (2022)	Empirical/descriptive AI-in-education study; Academic and administrative AI role data; source not reported in manuscript	not reported in manuscript	Academic/administrative AI roles; assessment and support practices	AI-integrated professional support; assessment/co-teaching context; adoption barriers; Not fully assessable; provisional lower tier
Simuţ et al. (2024)	Structural equation modeling study; Teacher competence modeling data; source not reported in manuscript	SEM	Teachers' competence; ethics/responsible AI use	Psychometric/explanatory; ethics-as-practice; Tier 1, moderate
Alwakid et al. (2025)	Empirical AI-and-competence study; Instructional planning and student-performance data; source not reported in manuscript	not reported in manuscript	Teacher competence in AI-supported instructional planning	AI-integrated PD/intervention evidence; assessment and human–AI co-teaching; Not fully assessable; provisional lower tier
Xu et al. (2025)	Framework/instrument-oriented empirical study; Technology-integration competence data; source not reported in manuscript	Content-based AI-inclusive framework/TPACK-derived analysis	Technology integration competence; AI-inclusive TPACK	Pedagogical AI competence; competence synthesis map; Tier 1/3, moderate
Lee and Bryan (2025)	Teacher-education/PD intervention study; AI integration in teacher education data; source not reported in manuscript	not reported in manuscript	Preservice teacher competence; AI literacy/integration	PD roadmap; foundational AI literacy and GenAI specificity; Tier 4, moderate-to-lower

The full row-level audit fields requested by reviewers—country or region, sample, data source, main findings, and reported limitations—are provided in Supplementary Table S1.

### 2.6 Quality, trustworthiness, and evidential-strength appraisal

Because the corpus combined psychometric validation studies, ML and LA studies, reviews, and professional-development interventions, an MMAT-informed, design-sensitive tiering approach was used rather than a single risk-of-bias checklist or a full MMAT score. The Mixed Methods Appraisal Tool (MMAT) informed the appraisal because it provides criteria for qualitative, quantitative, and mixed-methods empirical designs (Hong et al., 2018). However, the tier in Table 2 reflects the broad design class of a study, whereas the higher, moderate, or lower trustworthiness label reflects the reviewers' per-study judgement within that design class. For study types that were not fully captured by MMAT alone, the appraisal was supplemented with design-specific trustworthiness criteria aligned with the review questions. Psychometric studies were examined for construct definition, content development, sampling adequacy, reliability, EFA/CFA or SEM evidence, and criterion or invariance evidence where reported. ML and LA studies were examined for data-source transparency, feature justification, validation strategy, reporting of performance metrics, overfitting control, explainability, fairness, and external validation. Review papers were examined for search transparency, eligibility criteria, corpus accounting, and synthesis procedure. Professional-development studies were examined for intervention description, comparison or control condition, pre/post or follow-up measurement, attrition, and alignment between intervention content and competence outcomes.

**Table 2:** MMAT-informed, design-sensitive tiering used to interpret the reviewed corpus.

Design tier	Study type	Design-sensitive criteria informing per-study judgement	Interpretive use in this review
Tier 1	Psychometric validation and SEM studies	Explicit construct definition, item or content development, adequate sample, EFA/CFA or SEM, reliability, and criterion or invariance evidence where available	Stronger support for identifying competence dimensions and measurement structures; not treated as evidence of intervention effectiveness
Tier 2	ML and LA prediction or modeling studies	Transparent data sources and features, train/test split or cross-validation, reported performance metrics, overfitting checks, and attention to explainability, fairness, or external validation	Moderate support for analytic roles and methodological gaps; deployment claims remain tentative when construct validity, fairness, or external validation are absent
Tier 3	Systematic, scoping, or descriptive reviews and mapping studies	Explicit search or corpus strategy, eligibility criteria, transparent coding, and synthesis procedure	Useful for field-level patterns and gap identification; strength depends on search breadth and reproducibility
Tier 4	Professional-development interventions and quasi-experimental or pre/post studies	Clear intervention description, outcome alignment, comparison condition or baseline, follow-up, attrition reporting, and transfer evidence	Supports feasibility and design implications; causal claims are cautious when randomization, controls, or long-term follow-up are missing
Tier 5	Conceptual, case-based, or descriptive accounts	Clear theoretical rationale, context description, and connection to existing competence constructs	Used as contextual or theory-building evidence; not treated as independent empirical evidence of effectiveness or validated competence structure

Although quality appraisal is optional in scoping reviews under PRISMA-ScR and JBI guidance, it was conducted after study inclusion only to inform the interpretation of the findings and did not influence study inclusion or exclusion (Tricco et al., 2018; Peters et al., 2020). Each included article was appraised independently by two reviewers from the four-member team (B.S., Z.C., A.K., and L.S.). Per-study appraisals were recorded as higher, moderate, or lower trustworthiness for the purpose of narrative synthesis; these labels were not assigned mechanically from the design tier alone. Disagreements were first resolved through discussion within the reviewer pair; unresolved cases were adjudicated by N.B. No pooled quality score, meta-analytic certainty rating, or effect-size synthesis was calculated because the purpose of the review was to map a heterogeneous evidence base rather than estimate intervention effects. Table 2 should, therefore, be read as an interpretive weighting scheme for this review: tiers identify design classes, while the study-level higher/moderate/lower labels in the extraction matrix record the actual per-study trustworthiness judgements. It is not a universal hierarchy for all education research.

The synthesis strategy combined descriptive mapping and narrative analysis. Quantitative variables such as year, location, and journal were coded to show the distribution of the corpus. Qualitative data on methodology and competence concepts were examined through thematic analysis informed by Braun and Clarke (2006) and through comparative analysis across study roles and competence constructs. The studies were categorized using two organizing axes. On the first axis, the use of AI, ML, or LA was identified as an analytical tool, a dashboard or modeling framework, an object of competence, or professional learning support. On the second axis, competence emphasis was classified as technical and AI literacy, pedagogy, ethics, self-regulation, or professional development. This cross-tabulation supported the development of the AI-based teacher-competence synthesis map and the AI, ML, and LA method classification reported in Section 5.

The competence synthesis map was derived through a three-step coding procedure. First, all explicit competence labels, scale dimensions, subscales, and intervention targets were charted verbatim from each article. Second, conceptually similar labels were grouped through constant comparison; for example, AI knowledge, AI literacy, prompt awareness, and tool understanding were grouped only when the study treated them as basic conceptual or technical understanding. Third, the provisional groups were checked against the methodological role of AI in each study to avoid mixing competence content with analytic technique. Categories were retained when they appeared in multiple studies or represented a distinct theoretical construct, such as self-regulated AI learning or human–AI co-teaching. This procedure produced five strata: foundational AI literacy; pedagogical AI competence; ethical, critical, and reflective AI competence; AI-mediated self-regulated development; and assessment and human–AI co-teaching competence. The resulting synthesis should therefore be read as a structured map of the reviewed literature, not as a definitive hierarchy of teacher competence. The row-level grounding for each stratum and method role is shown explicitly in Supplementary Table S2.

## 3 Results

### 3.1 Descriptive characteristics of the corpus

**Figure 2:**
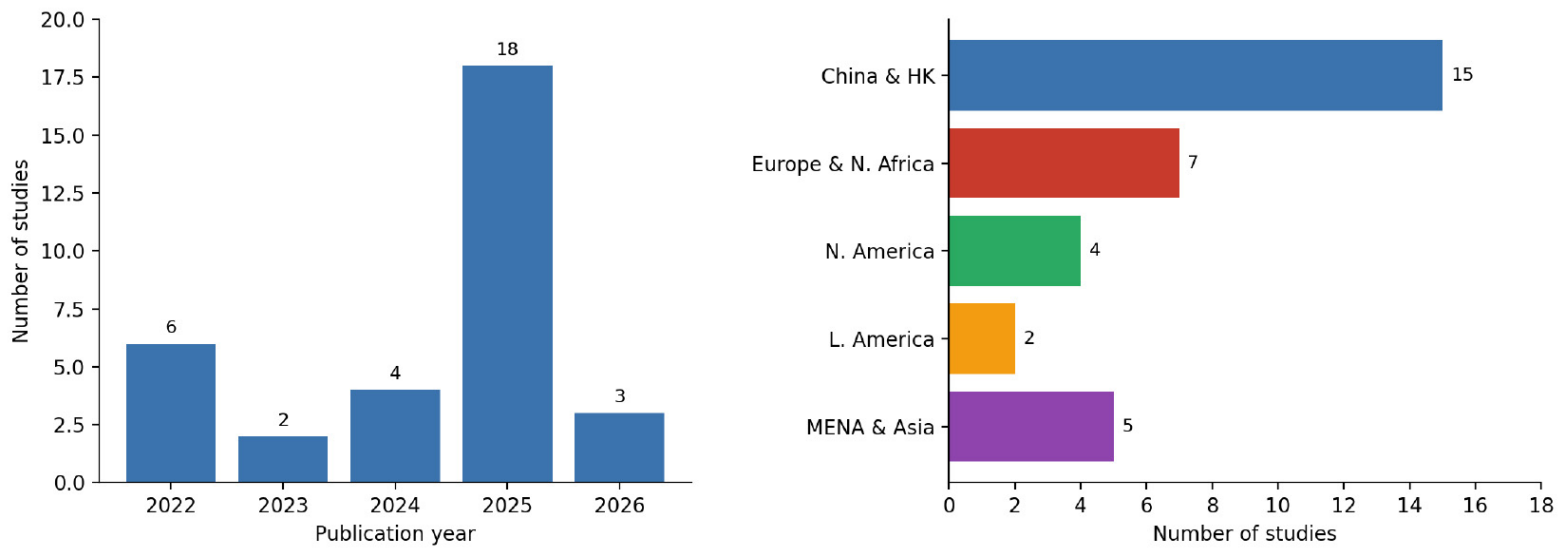
Publication years and regional distribution of studies.

The included corpus was recent and multidisciplinary, with studies published between 2022 and 2026 across education, educational technology, computer science, and engineering venues. The majority of articles were published after the emergence of generative AI in education, with the largest share published in 2025. Areas with strong research activity included China, Hong Kong, Europe, the United States, the Middle East, and Asia Pacific. Figure 2 provides detailed publication-year and geographical distributions.

### 3.2 AI, ML, and LA methods used for competence-related inference

AI, ML, and LA served four main functions in the reviewed corpus. Predictive-evaluative studies used machine learning to classify or estimate teaching quality and academic development. Rashid (2023) used accreditation data from 1,556 faculty members and reported higher predictive accuracy for *k*-means combined with binary logistic regression than for regular logistic regression alone. Zhong (2023) reported higher mean accuracy for GA-SVM than for SVM alone when modeling 16 teaching-quality parameters. Wu (2022) proposed a machine-learning-based competence model for primary and secondary teachers in online teaching, while Luo and Yang (2022) used CNN and LSTM methods with smart-classroom data to analyse teacher–student interaction quality. Zambrano-Romero et al. (2025) identified decision trees, SVM, neural networks, ensemble learning, and clustering as recurring techniques in teaching-quality assessment.

In the multilevel-explanatory role, ML was combined with hierarchical or regularized models to identify predictors of teacher competence. Cao et al. (2026) examined AI-TPACK among 2,166 teachers and reported AI-Ethics and AI-TCK as important teacher-level predictors, with school digital strategy acting as a contextual moderator. Yoon and Kim (2025) used LASSO to reduce 132 TALIS 2018 predictors to 16 variables related to PD practices, teacher characteristics, and organizational climate. These studies illustrate interpretive uses of ML, although they do not remove the need for construct validation.

In the descriptive-analytic role, LA was used to map competence patterns rather than to rank teacher performance. Yau et al. (2025) used Epistemic Network Analysis with interview transcripts from teachers in professional development to map links among TPACK components for AI teaching. Salas-Pilco et al. (2022) reviewed 30 studies on AI and LA in teacher education and identified LA dashboards, behavioral analytics, and digital literacy diagnostics as recurring approaches.

In the psychometric-validation role, AI was the object of competence measurement rather than the inference mechanism. This was the largest group of studies. Instruments such as T-GAIC (Shi, 2025), AI-SRLS (Zhang et al., 2025), AI-TPACK (An et al., 2026), TAICS (Chiu et al., 2025), Delcker et al.'s (2025) AI competence self-perception instrument, and RAIS (Ramazanoglu and Akın, 2025) were developed or validated with conventional psychometric procedures, including exploratory or confirmatory factor analysis, item analysis, and criterion-related validity testing. These studies do not use ML or LA as pipelines, but they provide measurement foundations that future analytics work could draw on.

Table 3 summarizes the method roles and cross-cutting generative-AI competence content. Because several studies contributed to more than one conceptual category, the categories are treated as non-exclusive synthesis roles rather than as mutually exclusive scoring bins.

**Table 3:** Analytic roles and cross-cutting competence content.

Category	Techniques/content	References
Predictive-evaluative	SVM and GA-SVM; CNN, LSTM; decision trees and ensembles; *k*-means; logistic regression; improved/hybrid ML	Rashid, 2023; Zhong, 2023; Luo and Yang, 2022; Wu, 2022; Zambrano-Romero et al., 2025
Multilevel-explanatory	Multilevel ML; LASSO regularized regression	Yoon and Kim, 2025; Cao et al., 2026
Descriptive-analytic	Epistemic Network Analysis; LA dashboards; behavioral and qualitative coding	Salas-Pilco et al., 2022; Lameras and Arnab, 2022; Yau et al., 2025; Ahmad et al., 2022
Psychometric-validation	EFA and CFA; Delphi panels; SEM and PLS SEM	Shi, 2025; Zhang et al., 2025; An et al., 2026; Chiu et al., 2025; Delcker et al., 2025; Ramazanoglu and Akın, 2025; Tan et al., 2025c; Simuţ et al., 2024; Alwakid et al., 2025; Xu et al., 2025
Generative-AI competence	ChatGPT and GenAI as workflow content; prompt engineering; output auditing; vibe-coding	Tan et al., 2025a; Shi, 2025; Elizondo-Garcia, 2026; Ding et al., 2024; Tan et al., 2025b; Alwakid et al., 2025; Lee and Bryan, 2025

### 3.3 Teacher competence dimensions and instruments

The reviewed papers used different labels for related competence areas. Recurring areas included pedagogical AI integration through AI-TPACK and i-TPACK (Cao et al., 2026; An et al., 2026; Tan et al., 2025b), AI literacy and readiness (Chiu et al., 2025; Ramazanoglu and Akın, 2025; Aravantinos et al., 2026), ethical and responsible AI use (Shi, 2025; Chiu et al., 2025; Brandão et al., 2024; Simuţ et al., 2024), AI-related assessment practices (Celik et al., 2022; Tan et al., 2025a; Ahmad et al., 2022), self-regulated AI learning (Zhang et al., 2025), technology integration (Xu et al., 2025; Lee and Bryan, 2025), and human–AI co-teaching (An et al., 2026). These areas are reported here descriptively; Section 5 later groups them into a smaller synthesis map to avoid treating every label as a separate validated construct.

Across these models, the main difference was not only terminology but also theoretical grounding. TPACK extensions emphasized pedagogical, technological, and content knowledge (An et al., 2026; Tan et al., 2025b); self-perception and readiness instruments drew on self-efficacy traditions (Chiu et al., 2025; Delcker et al., 2025; Ramazanoglu and Akın, 2025); T-GAIC foregrounded generative-AI practices such as prompt design and output evaluation (Shi, 2025; Elizondo-Garcia, 2026); and AI-SRLS focused on self-regulated professional learning (Zhang et al., 2025). No included study integrated all of these perspectives in a single validated instrument.

### 3.4 AI-integrated professional development and intervention evidence

Several studies treated AI as a practical support for teachers, including work on ChatGPT-supported instructional planning Alwakid et al. (2025), AI assistance with administrative tasks Ahmad et al. (2022), and AI affordances for teacher agency Lameras and Arnab (2022). These studies shift attention from individual knowledge alone to the professional contexts in which teachers use, interpret, and evaluate AI-supported tools.

### 3.5 Reported limitations and uncertainty across the evidence base

Three limitations recurred across the mapped evidence. First, several instrument-validation studies used adequate samples for EFA or CFA but were limited by country, language, or education level (Chiu et al., 2025; Ramazanoglu and Akın, 2025). Second, ML and LA studies often reported performance metrics without comparable detail on explainability, fairness, or construct validity (Zhong, 2023; Zambrano-Romero et al., 2025). Third, the review literature pointed to the continued use of self-report and quasi-experimental designs and to limited longitudinal evidence (Dogan et al., 2025; Salas-Pilco et al., 2022). These limitations are interpreted further in the Discussion and converted into future-research priorities in Section 6.

## 4 Discussion

### 4.1 From AI about teachers to AI with teachers

The distinction between *AI about teachers* and *AI with teachers* is a central conceptual contribution of this scoping review. Existing models usually define what teachers are expected to know or be able to do with AI. For example, AI-TPACK extends technological, pedagogical, and content knowledge into AI-rich instruction, TAICS emphasizes self-efficacy and responsible AI use, and T-GAIC focuses on generative-AI-specific capacities such as prompt design and output evaluation (Shi, 2025; An et al., 2026; Chiu et al., 2025). These models are useful for specifying competence domains, but they do not always make explicit whether AI is being used to analyse teachers or to support teachers' professional action. The AI about/with distinction makes that relationship visible.

The framework adds a relational dimension to the map of the reviewed corpus. In the *AI about teachers* orientation, AI, ML, or LA systems treat teachers as the unit of analysis: they classify teaching quality, predict competence indicators, cluster professional profiles, or infer development needs from behavioral and institutional data (Zhong, 2023; Zambrano-Romero et al., 2025). In the *AI with teachers* orientation, AI is positioned as part of teachers' professional agency: teachers use AI to plan instruction, assess learning, reflect on practice, evaluate outputs, and make ethical decisions in context (Tan et al., 2025b; Alwakid et al., 2025). The framework does not replace existing competence areas; instead, it provides a transferable analytic lens for describing how AI was positioned differently across the included studies.

This distinction helps interpret three patterns in the mapped literature. First, studies with similar labels, such as “AI competence” or “teacher analytics,” often used different evidence sources and validity standards. Second, in *AI about teachers* studies, high model accuracy was difficult to interpret when explainability, fairness checks, or links to teacher-competence theory were not reported. Third, *AI with teachers* studies suggest that future measurement may need to attend not only to technical skill but also to pedagogical judgement, ethical reasoning, and co-agency with AI tools. For example, Cao et al. (2026) used ML to predict AI-TPACK, Yoon and Kim (2025) used LASSO to identify professional development predictors, and Yau et al. (2025) used ENA to map AI teaching competence. Taken together, these examples indicate that AI was represented in the corpus both as a means of modeling teacher competence and as part of the competence being modeled.

A methodological implication concerns how validation arguments may need to be framed when AI, machine learning, and learning analytics are used for competence-related inference. When a study moves from descriptive mapping toward predictive modeling, reliability and internal consistency remain relevant, but transparency in decision-making and attention to possible consequences also become important.

### 4.2 Methodological strengths and weaknesses

Several methodological strengths were visible in the reviewed corpus, especially in psychometric studies. Instrument-development papers commonly used EFA and CFA procedures, reported internal-consistency evidence such as Cronbach's α, and compared proposed instruments with existing scales (Shi, 2025; Chiu et al., 2025; Ramazanoglu and Akın, 2025). Studies on machine learning in teaching evaluation and learning analytics reported accuracy, computational performance, or representations intended for practical use, such as the GA-SVM pipeline (Zhong, 2023) and CNN/LSTM (Luo and Yang, 2022) procedures. Multilevel and regularized ML approaches (Yoon and Kim, 2025; Cao et al., 2026) further illustrated how ML can be combined with hierarchical inference, although these examples do not by themselves establish general effectiveness or transferability.

Nevertheless, three recurring limitations were visible in the bounded corpus. First, explainability was often underreported: the reviewed ML and LA studies rarely explained which features drove competence predictions. The corpus itself does not establish SHAP, LIME, or counterfactual explanations as tested solutions in teacher-competence research; these methods are, therefore, presented here as author-proposed options for future work responding to the observed explainability gap (Wachter et al., 2018; Ribeiro et al., 2016; Lundberg and Lee, 2017). Second, fairness received limited explicit treatment; the high-performing ML pipelines or LA studies cited in the review (Zhong, 2023; Luo and Yang, 2022) did not report subgroup error analysis. Fairness-aware modeling is likewise proposed as a methodological extension rather than as a practice already demonstrated in the reviewed corpus. Third, the construct validity of ML or LA indicators remained difficult to judge when indicators were not clearly tied to defined competence constructs. For instance, 16 “evaluation indices” were applied in an SVM classifier (Zhong, 2023); the theoretical construct of those variables was seldom linked to any competence definition. Thus, the corpus supports the identification of validity, explainability, and fairness reporting gaps, while the specific technical remedies should be read as proposed directions for future research. The quality appraisal reinforced this interpretation: instrument-validation studies provided relatively stronger evidence for measurement structures, whereas ML, LA, and professional-development studies more often supported provisional analytic roles, feasibility claims, or research-gap identification rather than robust claims about effectiveness, fairness, or transferability.

### 4.3 Black-box teacher evaluation, professional rights, and trust

The most important ethical issue raised by the predictive-evaluative strand is not whether models such as Zhong's GA-SVM can be accurate, but whether high accuracy is sufficient when the object of prediction is teachers' professional practice. Zhong (2023) reported higher mean accuracy for a GA-SVM classifier than for SVM across 16 teaching-quality parameters, and similar predictive-evaluative studies used accreditation records, neural-network interaction models, or hybrid ML pipelines to estimate teacher development or teaching quality (Rashid, 2023; Luo and Yang, 2022; Wu, 2022; Zambrano-Romero et al., 2025). These studies make the black-box dilemma concrete: the better the model performs as a classifier, the more tempting it becomes for institutions to use it in appraisal, promotion, remediation, workload allocation, or professional-development triage, even when the reasons for a particular classification remain opaque.

In teacher assessment, opacity is pedagogically and ethically consequential. A score or risk label is not merely a technical output; it can shape a teacher's reputation, employment trajectory, access to professional learning, confidence, and sense of professional agency. If teachers cannot know which evidence was used, whether the evidence was valid, how features were weighted, or how errors can be corrected, algorithmic assessment weakens basic conditions of professional due process. The relevant “right to explanation” should, therefore, be understood not only as a demand for model transparency, but also as a procedural right to intelligible reasons, data correction, contextual response, contestability, and accountable human review (Mittelstadt et al., 2016; Kroll et al., 2017; Wachter et al., 2018). International AI-governance developments similarly treat education and employment-related AI uses as high-risk domains, reinforcing the need for documentation, human oversight, and contestability when AI systems materially affect professional opportunities (European Parliament and Council of the European Union, 2016).

This concern connects directly to the XAI and subgroup-fairness gaps identified in this review. Aggregate accuracy can conceal systematic errors for teachers in particular subjects, language groups, institutions, contract types, or career stages. A model that appears accurate overall could still over-identify novice teachers, teachers in under-resourced schools, minority-language teachers, or teachers whose pedagogies are less visible in the available data. For this reason, explainability and fairness should not be treated as optional technical enhancements added after deployment. In high-stakes teacher evaluation, they are part of the validity argument: a system should be able to show which pedagogically meaningful features influenced the output, how uncertain the output is, whether error rates differ across relevant subgroups, and who is responsible for reviewing contested cases.

The pedagogical issue is equally important. If explanations only identify statistical proxies, such as interaction frequency, dashboard activity, or generic evaluation indices, they may encourage performative compliance rather than professional growth. A teacher might learn to optimize visible indicators while the deeper qualities of teaching—diagnosis of student thinking, formative feedback, ethical judgement, classroom care, and adaptive pedagogy—remain undermeasured. Trustworthy AI-based assessment therefore requires a shift from black-box ranking toward interpretable, theory-grounded, and dialogic evaluation. The system should support professional learning by making evidence discussable, not replace professional judgement with an opaque score.

### 4.4 Ethics as teacher competence

A further pattern is that ethics and fairness appeared in two related ways in the reviewed studies: as properties of responsible AI application and as competence that teachers may need to develop. TAICS (Chiu et al., 2025), T-GAIC (Shi, 2025) and RAIS (Ramazanoglu and Akın, 2025) included ethics as an assessment area in relation to responsible AI application. Brandão et al. (2024) similarly treated ethics as part of AI education. Tan et al. (2025b) reported that teachers could discuss algorithmic bias conceptually but still had difficulty identifying ethical issues while applying AI in classroom activities. These findings suggest a gap between ethics as self-reported knowledge and ethics as situated professional judgement, but the corpus is not sufficient to determine how best to close that gap.

The black-box assessment problem extends this competence agenda. Teachers do not only need to use AI responsibly in their own lesson planning; they may also need the professional language to question AI systems used on them. Ethical AI competence should therefore include the ability to ask what construct a model claims to measure, what data are included or excluded, whether subgroup performance has been examined, what explanation is available, and how a teacher can contest or contextualize an output. In this sense, ethics is not only a content domain for teacher learning. It is also a condition of fair professional assessment.

### 4.5 Theory-driven feature design for interpretable ML pipelines

A concrete way to reduce the black-box risk is to connect ML pipelines with pedagogical and psychometric theory before modeling begins. Psychometric subscales offer one immediate bridge. Validated or emerging instruments such as TAICS, T-GAIC, AI-SRLS, AI-TPACK, and RAIS already organize teacher AI competence into named constructs, including AI knowledge, self-efficacy, readiness, ethical reasoning, pedagogical integration, self-regulated AI learning, and output evaluation (Shi, 2025; Zhang et al., 2025; An et al., 2026; Chiu et al., 2025; Ramazanoglu and Akın, 2025). Instead of feeding models only with unlabelled behavioral traces or broad evaluation indices, future pipelines could use these subscale scores as interpretable feature groups. A prediction or recommendation could then be traced to a construct such as pedagogical AI integration or ethical output auditing, rather than to an opaque composite. Because self-report data are vulnerable to bias, such features should be triangulated with observation, artifact, and professional-learning evidence rather than treated as sufficient on their own.

Three additional examples illustrate theory-driven, interpretable feature design. First, AI-TPACK or i-TPACK models could represent AI-TK, AI-PK, AI-TCK, AI-TPK, AI-PCK, and AI-TPCK as separate feature families, with interactions used only when they correspond to theorized relationships among technology, pedagogy, and content (Cao et al., 2026; An et al., 2026; Tan et al., 2025b). This would allow a model to distinguish limited technical familiarity from weak pedagogical integration, rather than producing a single undifferentiated competence score. Second, learning-analytics features could be mapped to self-regulated professional learning cycles: goal setting, strategy selection, prompt revision, output checking, reflection, and follow-up action (Zhang et al., 2025; Tan et al., 2025b). Such a design would make behavioral traces interpretable as evidence of professional learning processes rather than as generic activity counts. Third, classroom-observation, portfolio, or discourse features could be aligned with pedagogical rubrics, for example formative feedback, diagnostic questioning, differentiation, assessment alignment, and explicit teacher review of AI-generated outputs. ENA or similar network methods could then be used to show how teachers connect content, pedagogy, AI tools, and ethical reasoning in practice, instead of simply predicting a global quality label (Yau et al., 2025; Alwakid et al., 2025; Lee and Bryan, 2025).

These examples do not eliminate the need for validation or fairness analysis. They do, however, make the ML pipeline more contestable: features become inspectable, model explanations become pedagogically meaningful, and subgroup errors can be interpreted in relation to constructs rather than only to technical performance metrics. This is the point at which the review's integration gap, XAI gap, and fairness gap converge.

### 4.6 Geopolitical imbalance and governance transferability

The regional distribution of the included studies is itself a limitation of the evidence base. In the regional coding used for Figure 2, 15 of the 33 studies were associated with China and Hong Kong (45.5%), five with MENA and Asia (15.2%), seven with Europe and North Africa (21.2%), four with North America (12.1%), and two with Latin America (6.1%). If the China/Hong Kong and MENA/Asia categories are considered together, Asian or Asia-adjacent contexts account for 20 of the 33 studies (60.6%). This imbalance means that the review's synthesis is not geographically neutral. It may over-represent governance conditions, platform infrastructures, assessment cultures, and data-collection possibilities that are more compatible with centralized or system-level AI initiatives, while under-representing contexts where teacher evaluation and educational data governance are more locally fragmented.

This matters for transferability. Several predictive-evaluative studies in the corpus rely on institutional accreditation records, teaching-quality indicators, online-teaching traces, smart-classroom data, or school-level digital-strategy variables (Rashid, 2023; Zhong, 2023; Luo and Yang, 2022; Wu, 2022; Cao et al., 2026). Such pipelines are easiest to scale when a system can standardize data definitions, collect comparable teacher-level records, define common evaluation indicators, and connect analytics outputs to a relatively coherent teacher-development or accountability structure. In highly decentralized educational systems, such as many European and North American settings, these assumptions may not hold. Governance authority may be distributed across ministries, states, provinces, districts, municipalities, school boards, universities, unions, vendors, and individual institutions. Data-protection regimes such as the GDPR, together with the EU AI Act's high-risk treatment of education- and employment-related AI, also make lawful basis, purpose limitation, data minimization, human oversight, documentation, and contestability central design requirements rather than optional implementation details (European Parliament and Council of the European Union, 2016, 2024).

The centralized–decentralized distinction affects deployment in at least four ways. First, data ownership and stewardship differ: a ministry or national platform may be able to assemble a unified teacher dataset, whereas a decentralized system may require data-sharing agreements across districts, universities, platform vendors, and professional bodies. Second, standardization is harder when teacher-evaluation rubrics, professional standards, subject curricula, and observation instruments vary by jurisdiction. A model trained on one set of teaching-quality indices may therefore encode local policy assumptions rather than general teacher competence. Third, teacher-evaluation policy is not only technical but also professional and legal; in decentralized systems, the use of AI for appraisal may need negotiation with teacher associations, data-protection officers, institutional review bodies, and local governance boards. Fourth, interoperability becomes a scalability condition. Analytics models cannot transfer if learning-management systems, observation platforms, HR records, PD systems, and assessment databases use incompatible identifiers, metadata, and data-quality standards.

For centralized AI-driven models to transfer across governance contexts, future studies would need to test more than predictive accuracy. They would need to document data provenance, lawful data access, feature equivalence, measurement invariance, subgroup performance, local teacher-evaluation policy, and the availability of human review and appeals. In decentralized systems, transfer may require federated or privacy-preserving analytics, interoperable data standards, locally validated competence constructs, and modular models that can be recalibrated without assuming a single national data infrastructure. This governance-transferability analysis is a substantive contribution of the review because it specifies why teacher-competence analytics cannot be evaluated apart from the policy and data infrastructures in which they operate. The point is not to rank governance regimes, but to identify a geopolitical and methodological research gap: the field needs cross-context comparative studies that deliberately compare centralized and decentralized systems, include under-represented regions, and examine whether AI-based teacher-competence models remain valid, fair, explainable, and professionally acceptable when moved across governance regimes.

### 4.7 Adoption barriers and contextual factors

Reported barriers to AI adoption were grouped into four categories within this review. Institutional barriers included lack of access to digital tools and strict schedules (Xiao et al., 2025; Ahmad et al., 2022). Individual barriers were associated with low AI preparedness, fear of AI, and perceptions of AI complexity (Delcker et al., 2025; Ramazanoglu and Akın, 2025). Pedagogical barriers concerned uncertainty about how to integrate AI into subject-matter teaching (Ding et al., 2024; Alwakid et al., 2025; Lee and Bryan, 2025). Ethical and legal barriers included privacy, data governance, and bias-related concerns (Brandão et al., 2024; Aravantinos et al., 2026; Simuţ et al., 2024). Xiao et al. (2025) suggested that a hybrid-flexible delivery format may help address some institutional constraints through flexible training options, but this does not by itself resolve pedagogical or ethical concerns. Similarly, Aravantinos et al. (2026) indicated that technical training alone may be insufficient.

### 4.8 Educational implications

For teacher educators, the mapped literature supports caution about treating AI skills as a standalone curriculum component. In this corpus, AI competence was more often framed as pedagogically situated through case-based learning (Ding et al., 2024), empirical experimentation (Brandão et al., 2024; Lee and Bryan, 2025), and intelligent-TPACK scaffolding (Tan et al., 2025b), rather than as declarative tool knowledge alone. For school leaders, the multilevel findings (Yoon and Kim, 2025; Cao et al., 2026) identify school digital strategy and PD design as relevant contextual conditions for teacher AI competence, although causal or policy conclusions remain beyond the evidence reviewed here. For researchers, the review offers a practical design principle: future ML/LA inference should be linked explicitly to validated competence constructs and to the governance conditions under which teacher data are collected and used.

## 5 Proposed synthesis

From this bounded scoping analysis, five interrelated synthesis outputs have been developed: (i) the *AI about teachers*/*AI with teachers* framework; (ii) a competence synthesis map; (iii) a method-role classification for AI, ML, and LA; (iv) competence mapping; and (v) a research-gap and professional-development roadmap. The outputs are outlined as conceptual frameworks rather than checklist models; they are based on the reviewed literature and are meant to inform future studies, LA designs, and professional development designs. Their evidential status is uneven and should be interpreted through the appraisal scheme in Table 2: constructs supported by psychometric validation provide stronger evidence for measurement structure, whereas professional-development roadmaps, human–AI co-teaching claims, fairness-aware analytics, and longitudinal-impact claims remain more provisional. The synthesis aims to extend existing teacher-AI competence models by linking competence content to the role of AI in the research design: in the mapped studies, AI was positioned as an evaluator of teachers, an analytic model of teacher development, a professional tool used by teachers, or a co-teaching partner. The subsections below describe the competence synthesis map and the method classification separately to avoid implying that the map is a validated hierarchy.

### 5.1 AI-based teacher competence synthesis map

The synthesis map consolidates the overlapping instruments into five interrelated competence strata. It was not generated from a single pre-existing model; rather, it was constructed by comparing the extracted competence labels, measurement dimensions, and intervention targets across the reviewed studies. The strata, therefore, represent recurring clusters in the corpus rather than mutually exclusive psychological traits.

(i) Foundational AI Literacy covers conceptual understanding of AI, ML, LA, data, and generative models. Examples include T-GAIC dimensions related to basic generative-AI understanding and TAICS dimensions on AI knowledge and self-efficacy (Shi, 2025; Chiu et al., 2025). (ii) Pedagogical AI Competence refers to the AI-TPACK family: AI-TK, AI-PK, AI-PCK, AI-TPK, AI-TCK, and AI-TPCK, including human-teacher and machine-teacher dimensions. Examples include An et al.'s (2026) AI-TPACK scale and Tan et al.'s (2025b) i-TPACK professional development intervention. (iii) Ethical, Critical, and Reflective AI Competence includes ethical use, bias detection, accountability, and reflective practice. This category was assigned to studies that treated ethics as a competence dimension rather than only as a general concern, such as TAICS and the review of AI ethics education by Chiu et al. (2025); Brandão et al. (2024). (iv) AI-Mediated Self-Regulated Development covers metacognitive control of AI-driven learning, professional engagement, and ongoing competence renewal. Zhang et al.'s (2025) AI-SRLS scale and Tan et al.'s (2025b) longitudinal professional learning design are representative examples. (v) Assessment and Human–AI Co-Teaching Competence covers AI-augmented assessment, automated feedback, and co-instruction between teachers and AI systems. Examples include AI-TPACK work that distinguishes human- and machine-teacher roles and studies on AI-supported academic or administrative assessment practices (An et al., 2026; Ahmad et al., 2022).

This coding process also explains why some studies appear in more than one stratum. For instance, TAICS contributes to foundational literacy, ethics, and readiness, while AI-TPACK studies contribute to pedagogical competence and human–AI co-teaching. Such overlap was treated as evidence of multidimensionality rather than as a coding error.

### 5.2 AI, ML, and LA method classification

The method classification summarizes the same roles reported in Table 3, but here it is used to interpret how AI relates to teachers. Predictive-evaluative and multilevel-explanatory studies position AI mainly as an analytic lens on teachers, whereas descriptive-analytic and psychometric-validation studies more often map competence constructs or measurement structures. The generative AI category cuts across these roles because prompt design, output auditing, workflow integration, and AI-assisted code generation are treated as emerging content areas for teacher competence (Shi, 2025; Tan et al., 2025b).

### 5.3 Teacher competence mapping

The mapping links each competence stratum to its dominant operationalization in the reviewed corpus. Foundational AI literacy was often operationalized through self-report scales validated using EFA and CFA. Pedagogical AI competence was captured through TPACK-extension instruments and, in some studies, modelled using ML over multilevel data. Ethical and critical competence typically appeared as a subscale rather than as an independently validated construct. Self-regulated AI learning was operationalized through AI-SRLS and emerging metacognitive instruments. Assessment and human–AI co-teaching competence was represented through studies on automated assessment, feedback, and co-instruction. This mapping suggests that the evidentiary base remains uneven: pedagogical and literacy strata are somewhat more directly supported by validation studies, whereas ethical, self-regulatory, and co-teaching strata should be treated as emerging and would benefit from further measurement development, cross-context validation, and stronger intervention evidence.

### 5.4 Research gaps and roadmap

Eight gaps are identifiable within the bounded corpus. Some gaps are directly observed in the reviewed studies: limited cross-cultural and longitudinal validation, limited explainability reporting in ML and LA teaching evaluation, absence of reported subgroup error analyses, weak links between competence models and ML or LA inference pipelines, and scarcity of experimental PD studies incorporating control groups, randomization, and competence transfer measurement. Additional author-identified priorities, informed by corpus patterns, concern ethics-as-practice, regional balance, and GenAI-specific measurement. Section 6 therefore separates evidence-derived gaps from the authors' proposed methodological agenda for addressing them.

A tentative four-phase PD roadmap is proposed from the intervention and professional-learning evidence, rather than established as a tested sequence. (R1) Diagnosis could use validated competence instruments (Chiu et al., 2025; Ramazanoglu and Akın, 2025). (R2) Foundational AI literacy could be developed through case-based and hands-on experiences (Ding et al., 2024; Brandão et al., 2024; Lee and Bryan, 2025). (R3) Pedagogical integration could use i-TPACK or AI-TPACK scaffolds (Li et al., 2025; Tan et al., 2025b,c). (R4) Reflective and ethical consolidation could involve embodied simulation and longitudinal coaching (Xiao et al., 2025; Tan et al., 2025b; Simuţ et al., 2024). The roadmap should be treated as iterative and provisional, because repeated diagnosis may be needed as AI tools continue to evolve.

## 6 Future research directions

Future studies could adopt a broader array of information sources in teacher competence analytics, which may make findings from machine learning and LA studies easier to interpret. This recommendation follows from the corpus, where ML and LA approaches primarily utilized numeric, textual, and dashboard-type indicators (Zhong, 2023; Yoon and Kim, 2025; Wu, 2022). By contrast, the use of multimodal classroom assessment, including audio, visual, gesture, and eye-tracking information, is an author-proposed extension rather than a conclusion directly demonstrated by the reviewed studies. Such multimodal data might offer a richer depiction of teachers' pedagogical and reflective competence, but would require careful alignment, annotation, consent, and governance processes (Luo and Yang, 2022; Salas-Pilco et al., 2022). Similarly, feature-level explanation, uncertainty reporting, and counterfactual analysis are proposed methodological safeguards for future work, not established practices in the reviewed corpus.

In future research, greater attention could be directed toward fairness, privacy, and validity across contexts. The corpus motivates this recommendation because high-accuracy teacher-evaluation models generally did not report subgroup error analyses (Rashid, 2023; Zhong, 2023). The stronger proposal that future studies assess outcomes by gender, language, subject area, or institution type reflects the authors' methodological agenda for high-stakes teacher competence analytics. The measurement invariance assessment done for TAICS (Chiu et al., 2025) serves as an example from psychometric validation, although comparable checks have not yet been systematically applied to ML and LA systems in this corpus.

Adaptive PD, longitudinal analysis, generative AI, and regional balance are areas where the corpus provides partial but incomplete evidence. LASSO, multilevel machine learning, and teacher-facing LA show how models might support competence-sensitive PD, but their use for highly personalized PD remains a proposed extension (Yoon and Kim, 2025; Cao et al., 2026). Longitudinal research would also be useful because current intervention evidence gives only limited insight into whether competence develops linearly, abruptly, or nonlinearly over time (Tan et al., 2025b). Prompt engineering, output auditing, and AI-assisted code generation are visible in recent studies, but their measurement remains underdeveloped (Shi, 2025; Tan et al., 2025b). Finally, because the existing corpus is geographically uneven and concentrated in China/Hong Kong and MENA/Asia-coded contexts, broader comparative evidence from Africa, South Asia, Latin America, Europe, and North America is proposed as a priority for future reviews and empirical studies (Zambrano-Romero et al., 2025; Salas-Pilco et al., 2022). Table 4 consolidates these evidence-derived limitations and author-proposed opportunities.

**Table 4:** Research gaps and future research opportunities identified in the reviewed literature.

Gap	Current limitation	Research opportunity	References
Validity	Instruments validated in one country, language, or educational level	Cross-cultural replication and measurement invariance testing	Shi, 2025; Zhang et al., 2025; An et al., 2026; Chiu et al., 2025; Delcker et al., 2025; Ramazanoglu and Akın, 2025; Xu et al., 2025
Explainability	Reviewed high-accuracy ML models rarely report explainable AI (XAI)	Proposed use of SHAP, LIME, and counterfactual reporting	Zhong, 2023; Luo and Yang, 2022; Wu, 2022; Zambrano-Romero et al., 2025
Fairness	No reported subgroup error analyses in ML and learning analytics pipelines	Proposed subgroup evaluation and fairness-aware analytics	Rashid, 2023; Zhong, 2023; Luo and Yang, 2022; Wu, 2022
Integration	ML and learning analytics inputs are often weakly connected to competence theory	Joint psychometric and ML analytics modeling	Zhong, 2023; Yoon and Kim, 2025; Wu, 2022; Cao et al., 2026
Evidence base	Cross-sectional self-report studies are common; few randomized controlled trials	Cluster-randomized trials and longitudinal growth models	Celik et al., 2022; Li et al., 2025; Xiao et al., 2025; Tan et al., 2025b; Dogan et al., 2025; Salas-Pilco et al., 2022
Ethics-as-practice	Ethics is often measured through self-report Likert subscales	Embodied simulation and situated ethical judgement	Chiu et al., 2025; Tan et al., 2025b; Brandão et al., 2024; Simuţ et al., 2024
Regional balance	China/Hong Kong and MENA/Asia-coded contexts account for 20 of 33 studies	Comparative studies across centralized and decentralized governance systems	Zambrano-Romero et al., 2025; Fakhar et al., 2024; Salas-Pilco et al., 2022; European Parliament and Council of the European Union, 2016; 2024
GenAI specificity	General AI scales only partly capture GenAI-related competence	Development of dedicated GenAI competence instruments	Shi, 2025; Elizondo-Garcia, 2026; Tan et al., 2025b; Brandão et al., 2024; Lee and Bryan, 2025

## 7 Conclusions

This scoping review mapped 33 Web of Science-indexed papers published between 2022 and 2026 and identified a developing body of scholarship in which artificial intelligence, machine learning, and LA appear in two related roles: as tools for measuring or modeling teacher competence, and as part of the competence that teachers are expected to develop. Within this bounded corpus, teacher competence was discussed in relation to AI literacy, ethical reasoning, generative AI fluency, self-directed AI learning, and teacher–AI collaboration. The quality appraisal suggests that the methodological base remains uneven. Psychometric validation, supervised ML, regression, and LA are visible, but cross-cultural validation, interpretability, subgroup fairness analysis, and long-term evidence remain limited; therefore, the strongest conclusions concern mapped constructs and methodological gaps, not causal effectiveness or readiness for high-stakes implementation.

The review was not prospectively registered in a public registry, which limits transparency regarding protocol development. Although NTC experts provided domain-specific input during the review process, no external stakeholder consultation with teachers, school leaders, policymakers, or professional associations was conducted.

The five synthesis outputs—the *AI about teachers*/*AI with teachers* framework, competence synthesis map, method-role classification, competence mapping, and research-gap and professional-development roadmap—map the reviewed corpus rather than prove that particular AI-based approaches are effective across settings. Within that boundary, they make two clear contributions: they provide a relational framework for distinguishing AI systems that analyse teachers from AI systems that support teachers' professional agency, and they identify governance transferability as a central condition for interpreting teacher-competence analytics across contexts. In line with the increasing influence of generative AI on educational practice, one priority for future research is to develop and test teacher competence measures and professional-development designs that are pedagogically grounded, ethically informed, transparent, and sensitive to context.

## Data Availability

The original contributions presented in the study are included in the article/Supplementary material, further inquiries can be directed to the corresponding author.

## References

[B1] Ahmad S. F. Alam M. M. Rahmat M. K. Mubarik M. S. Hyder S. I. (2022). Academic and administrative role of artificial intelligence in education. Sustainability 14:1101. doi: 10.3390/su14031101

[B2] Almubarak A. Alhalabi W. Albidewi I. Alharbi E. (2025). An AI-powered framework for assessing teacher performance in classroom interactions: a deep learning approach. Front. Artif. Intell. 8:1553051. doi: 10.3389/frai.2025.1553051 40969168 PMC12442429

[B3] Alwakid W. N. Dahri N. A. Humayun M. Alwakid G. N. (2025). Exploring the role of AI and teacher competencies on instructional planning and student performance in an outcome-based education system. Systems 13:517. doi: 10.3390/systems13070517

[B4] An X. Bai J. Li Y. Zhou Y. Chai C. S. Hong J.-C. (2026). Development of an artificial intelligence technology pedagogical content knowledge (AI-TPACK) scale for human and AI concurrently as teachers. Asia Pac. Educ. Res. 35, 483–493. doi: 10.1007/s40299-025-01045-2

[B5] Aravantinos S. Lavidas K. Komis V. Karalis T. Papadakis S. (2026). Artificial intelligence in K-12 education: a systematic review of teachers' professional development needs for AI integration. Computers 15:49. doi: 10.3390/computers15010049

[B6] Arksey H. O'Malley L. (2005). Scoping studies: towards a methodological framework. Int. J. Soc. Res. Methodol. 8, 19–32. doi: 10.1080/1364557032000119616

[B7] Brandão A. Pedro L. Zagalo N. (2024). Teacher professional development for a future with generative artificial intelligence-an integrative literature review. Digit. Educ. Rev. 45, 151–157. doi: 10.1344/der.2024.45.151-157

[B8] Braun V. Clarke V. (2006). Using thematic analysis in psychology. Qual. Res. Psychol. 3, 77–101. doi: 10.1191/1478088706qp063oa

[B9] Cao X. Huang Z. Li M. He T. (2026). Teachers' AI-TPACK as a tangible outcome in the digital transformation of education: a machine learning-based multilevel approach. Teach. Teach. Educ. 169:105270. doi: 10.1016/j.tate.2025.105270

[B10] Celik I. Dindar M. Muukkonen H. Järvelä S. (2022). The promises and challenges of artificial intelligence for teachers: a systematic review of research. TechTrends 66, 616–630. doi: 10.1007/s11528-022-00715-y

[B11] Chiu T. K. F. Ahmad Z. Çoban M. (2025). Development and validation of teacher artificial intelligence (AI) competence self-efficacy (TAICS) scale. Educ. Inf. Technol. 30, 6667–6685. doi: 10.1007/s10639-024-13094-z

[B12] Delcker J. Heil J. Ifenthaler D. (2025). Evidence-based development of an instrument for the assessment of teachers' self-perceptions of their artificial intelligence competence. Educ. Technol. Res. Dev. 73, 115–133. doi: 10.1007/s11423-024-10418-1

[B13] Ding A.-C. E. Shi L. Yang H. Choi I. (2024). Enhancing teacher AI literacy and integration through different types of cases in teacher professional development. Comput. Educ. Open 6:100178. doi: 10.1016/j.caeo.2024.100178

[B14] Dogan S. Nalbantoglu U. Y. Celik I. Dogan N. A. (2025). Artificial intelligence professional development: a systematic review of TPACK, designs, and effects for teacher learning. Prof. Dev. Educ. 51, 519–546. doi: 10.1080/19415257.2025.2454457

[B15] Elizondo-Garcia J. (2026). Vibe-coding as a teaching competency in the use of artificial intelligence for medical education: a scoping review. Rev. Espanol. Educ. Med. 2:705121. doi: 10.6018/edumed.705121

[B16] European Parliament and Council of the European Union (2016). Regulation (EU) 2016/679 of the European parliament and of the council of 27 April 2016 on the protection of natural persons with regard to the processing of personal data and on the free movement of such data (General Data Protection Regulation). Off. J. Eur. Union L119, 1–88.

[B17] European Parliament and Council of the European Union (2024). Regulation (EU) 2024/1689 of the European parliament and of the council of 13 June 2024 laying down harmonised rules on artificial intelligence (Artificial Intelligence Act). Off. J. Eur. Union.

[B18] Fakhar H. Lamrabet M. Echantoufi N. El Khattabi K. Ajana L. (2024). Towards a new artificial intelligence-based framework for teachers' online continuous professional development programs: systematic review. Int. J. Adv. Comput. Sci. Appl. 15. doi: 10.14569/IJACSA.2024.0150450

[B19] Hong Q. N. Fábregues S. Bartlett G. Boardman F. Cargo M. Dagenais P. . (2018). The mixed methods appraisal tool (MMAT) version 2018 for information professionals and researchers. Educ. Inf. 34, 285–291. doi: 10.3233/EFI-180221

[B20] Kroll J. A. Huey J. Barocas S. Felten E. W. Reidenberg J. R. Robinson D. G. . (2017). Accountable algorithms. Univ. PA Law Rev. 165, 633–705.

[B21] Lameras P. Arnab S. (2022). Power to the teachers: an exploratory review on artificial intelligence in education. Information 13:14. doi: 10.3390/info13010014

[B22] Lee H. Bryan L. M. (2025). Integrating AI in teacher education: exploring the impact on preservice teacher competencies. Prof. Dev. Educ. 51, 478–494. doi: 10.1080/19415257.2025.2490000

[B23] Levac D. Colquhoun H. O'Brien K. K. (2010). Scoping studies: advancing the methodology. Implement. Sci. 5:69. doi: 10.1186/1748-5908-5-69 20854677 PMC2954944

[B24] Li K. Wang P. Chen G. (2025). How can AI be integrated into teacher professional development programs? A systematic review based on an adapted technology-based learning model. Teach. Teach. Educ. 168:105219. doi: 10.1016/j.tate.2025.105219

[B25] Lundberg S. M. Lee S. I. (2017). A unified approach to interpreting model predictions. Adv. Neural Inf. Process. Syst. 30, 4765–4774.

[B26] Luo Q. Yang J. (2022). The artificial intelligence and neural network in teaching. Comput. Intell. Neurosci. 2022:1778562. doi: 10.1155/2022/1778562 35720947 PMC9205693

[B27] Mittelstadt B. D. Allo P. Taddeo M Wachter S. Floridi L. (2016). The ethics of algorithms: mapping the debate. Big Data Soc. 3:2053951716679679. doi: 10.1177/2053951716679679

[B28] Peters M. D. J. Marnie C. Tricco A. C. Pollock D. Munn Z. Alexander L. . (2020). Updated methodological guidance for the conduct of scoping reviews. JBI Evid. Synth. 18, 2119–2126. doi: 10.11124/JBIES-20-00167 33038124

[B29] Ramazanoglu M. Akın T. (2025). AI readiness scale for teachers: development and validation. Educ. Inf. Technol. 30, 6869–6897. doi: 10.1007/s10639-024-13087-y

[B30] Rashid A. S. K. (2023). The extent of the teacher academic development from the accreditation evaluation system perspective using machine learning. J. Exp. Theor. Artif. Intell. 35, 535–555. doi: 10.1080/0952813X.2021.1960635

[B31] Ribeiro M. T. Singh S. Guestrin C. (2016). ““Why should I trust you?” Explaining the predictions of any classifier,” in *Proceedings of the 22nd ACM SIGKDD international conference on knowledge discovery and data mining* (New York, NY: Association for Computing Machinery), 1135–1144. doi: 10.1145/2939672.2939778

[B32] Salas-Pilco S. Z. Xiao K. Hu X. (2022). Artificial intelligence and learning analytics in teacher education: a systematic review. Educ. Sci. 12:569. doi: 10.3390/educsci12080569

[B33] Shi L. (2025). Assessing teachers' generative artificial intelligence competencies: instrument development and validation. Educ. Inf. Technol. 30, 23365–23384. doi: 10.1007/s10639-025-13684-5

[B34] Simuţ R. Simuţ C. Bădulescu D. Bădulescu A. (2024). Artificial intelligence and the modelling of teachers' competencies. Amfiteatru Econ. 26, 181–200. doi: 10.24818/EA/2024/65/181

[B35] Tammets K. Ley T. (2023). Integrating AI tools in teacher professional learning: a conceptual model and illustrative case. Front. Artif. Intell. 6:1255089. doi: 10.3389/frai.2023.1255089 38130325 PMC10733452

[B36] Tan X. Cheng G. Ling M. H. (2025a). Artificial intelligence in teaching and teacher professional development: a systematic review. Comput. Educ. Artif. Intell. 8:100355. doi: 10.1016/j.caeai.2024.100355

[B37] Tan X. Cheng G. Ling M. H. (2025b). Enhancing teachers' AI competency: a professional development intervention study based on intelligent-TPACK framework. Comput. Educ. Artif. Intell. 9:100521. doi: 10.1016/j.caeai.2025.100521

[B38] Tan X. Cheng G. Ling M. H. (2025c). Investigating the mediating role of TPACK on teachers' AI competency and their teaching performance in higher education. Comput. Educ. Artif. Intell. 9:100461. doi: 10.1016/j.caeai.2025.100461

[B39] Tricco A. C. Lillie E. Zarin W. O'Brien K. K. Colquhoun H. Levac D. . (2018). PRISMA extension for scoping reviews (PRISMA-ScR): checklist and explanation. Ann. Intern. Med. 169, 467–473. doi: 10.7326/M18-0850 30178033

[B40] Wachter S. Mittelstadt B. Russell C. (2018). Counter factual explanations without opening the blackbox: automated decisions and the GDPR. Harv. J. Law Technol. 31, 841–887.

[B41] Wu C. Zhang W. Hu L. Li M. (2026). Research on middle school teachers' technostress empowered by artificial intelligence. Front. Artif. Intell. 8:1732088. doi: 10.3389/frai.2025.1732088 41626577 PMC12852380

[B42] Wu J. (2022). Construction of primary and secondary school teachers' competency model based on improved machine learning algorithm. Math. Probl. Eng. 2022:6439092. doi: 10.1155/2022/6439092

[B43] Xiao J. Yang Y. Li M. (2025). Empirical study on the feasibility of hybrid-flexible training model for developing teachers' artificial intelligence competence. Educ. Inf. Technol. 30, 16835–16860. doi: 10.1007/s10639-025-13460-5

[B44] Xu G. Yu A. Xu C. Liu X. Trainin G. (2025). Investigating pre-service TCSL teachers' technology integration competency through a content-based AI-inclusive framework. Educ. Inf. Technol. 30, 4349–4380. doi: 10.1007/s10639-024-12982-8

[B45] Yau K. W. Dong T. Chai C. S. Chiu T. K. F. Meng H. King I. . (2025). Epistemic network analysis of in-service teachers' competency to teach artificial intelligence for secondary education. Comput. Educ. Artif. Intell. 9:100520. doi: 10.1016/j.caeai.2025.100520

[B46] Yoon I. Kim M. (2025). Determinants of teachers' positive perception on their professional development experience: an application of LASSO-based machine learning approach. Prof. Dev. Educ. 51, 875–889. doi: 10.1080/19415257.2023.2264296

[B47] Zambrano-Romero W. Rodriguez C. Pita-Valencia J. Zambrano-Romero W. J. Moran-Tubay J. M. (2025). Machine learning in the teaching quality of university teachers: systematic review of the literature 2014–2024. Information 16:181. doi: 10.3390/info16030181

[B48] Zhang W. Ning Y. Yu N. Yao D. Wu H. Wu J. . (2025). Development and validation of the artificial intelligence self-regulated learning scale (AI-SRLS) for teachers. Educ. Inf. Technol. 30, 22989–23013. doi: 10.1007/s10639-025-13670-x

[B49] Zhong Y. (2023). Evaluation and analysis of teaching quality of university teachers using machine learning algorithms. J. Intell. Syst. 32:20220204. doi: 10.1515/jisys-2022-0204

