# Supplementary material for: Artificial intelligence and teacher competence: a scoping review of assessment, analytics, and professional development
Source: Front Artif Intell. 2026 Jul 27;9:1901449. doi: 10.3389/frai.2026.1901449 (PMC13454033; doi:10.3389/frai.2026.1901449)
Supplement: Supplementary file 1 [file Data_Sheet_1.pdf]

Supplementary Table S1. Data-Extraction Matrix for the 33 Included Studies

This matrix makes the study-level coding used in the scoping review auditable. It provides the full row-level fields condensed from Table 1 in the manuscript, including country or region, sample, data source, main findings, reported limitations, and quality notes. Fields were extracted from the manuscript text, the reference-list metadata, and the study details already reported in the review. Where a field was not available in those materials, it is marked as "not reported in manuscript" rather than inferred.

Table 1: Data-extraction matrix for the 33 included studies.

Author & year	Country/region	Study type	Sample	Data source	AI/ML/LA method used	Competence dimension examined	Role of the method in synthesis	Main findings	Limitations	Quality assessment
Celik et al. (2022; ref1)	not reported in manuscript	Systematic review	not reported in manuscript	Published AI-for-teachers literature	Systematic review / narrative synthesis	Promises, challenges, and teacher-facing AI competence	Evidence base; assessment and AI-with-teachers context	Mapped promises and challenges of AI for teachers; used to frame AI-supported assessment and teacher professional roles.	Corpus count and study-level appraisal not reported in manuscript.	Tier 3, moderate; review transparency not fully auditable from manuscript.
Tan et al. (2025; ref2)	not reported in manuscript	Systematic review	not reported in manuscript	AI in teaching and teacher PD literature	Systematic review	AI in teaching and teacher professional development	Evidence base; PD and AI-with-teachers context	Synthesised AI in teaching and teacher PD; supports claim that AI competence is linked to teaching and professional learning.	Specific corpus details not reported in manuscript.	Tier 3, moderate; review design but details not fully reported in manuscript.
Li et al. (2025; ref3)	not reported in manuscript	Systematic review	not reported in manuscript	AI integration in teacher PD literature	Systematic review using adapted technology-based learning model	AI integration in teacher PD; pedagogical AI competence	PD roadmap; pedagogical integration	Used to support AI integration into PD and the need for pedagogically designed AI learning models.	Study count and appraisal details not reported in manuscript.	Tier 3, moderate; review/mapping evidence.
Rashid (2023; ref4)	Iraq/Kurdistan Region; University of Sulaimani	ML-based predictive/evaluative study	1,556 faculty members	Accreditation evaluation records	k-means clustering and binary logistic regression	Teacher academic development from accreditation perspective	Predictive-evaluative; AI about teachers	k-means plus binary logistic regression was reported as 90% more accurate than regular logistic regression for predicting development-related outcomes.	Explainability, subgroup fairness, and external validation not reported in manuscript.	Tier 2, moderate; large institutional dataset but limited construct/fairness reporting.
Zhong (2023; ref5)	not reported in manuscript	ML-based teaching-quality evaluation	not reported in manuscript	16 teaching-quality assessment parameters	GA-SVM compared with SVM	Teaching quality / teacher evaluation	Predictive-evaluative; AI about teachers	GA-SVM mean accuracy 98.36% versus 86.72% for SVM; training and testing times were 23.21 ms and 7.25 ms.	Theoretical link between 16 indices and teacher competence, explainability, fairness, and external validation were limited or not reported.	Tier 2, moderate-to-lower; strong performance reporting but weak construct and fairness transparency.
Yoon and Kim (2025; ref6)	not reported in manuscript	ML explanatory study	2,418 teachers	TALIS 2018 dataset	LASSO-based machine learning	Teachers' positive perceptions of PD experience	Multilevel/explanatory; AI about teacher development	Reduced 132 predictors to 16, including PD practices, teacher characteristics, and organisational climate.	Observational design; causal inference, external validation, and fairness checks not reported in manuscript.	Tier 2, moderate; large secondary dataset with regularised modelling.
Luo and Yang (2022; ref7)	not reported in manuscript	Neural-network teaching study	not reported in manuscript	Smart classroom interaction data	CNN and LSTM neural networks	Teacher–student interaction quality / teaching quality	Predictive-evaluative; AI about teachers	Used CNN/LSTM with smart classroom data to measure teacher–student interaction quality.	Sample, construct validation, explainability, fairness, and external validation not reported in manuscript.	Tier 2, lower-to-moderate; computational modelling but limited trustworthiness detail.
Wu (2022; ref8)	not reported in manuscript	ML competence-model study	Primary and secondary teachers; number not reported in manuscript	Online teaching environment indicators	Improved machine-learning algorithm	Primary and secondary teachers' competence model	Predictive/evaluative and integration-gap evidence	Constructed a competence model for teachers working in online teaching environments.	Sample size, feature validity, explainability, fairness, and external validation not reported in manuscript.	Tier 2, lower-to-moderate; method relevant but reporting limited in manuscript.
Cao et al. (2026; ref9)	not reported in manuscript	ML-based multilevel study	2,166 teachers	Teacher-level AI-TPACK and school-level digital-strategy data	Machine learning with multilevel modelling	AI-TPACK; AI ethics; AI-TCK; school digital strategy	Multilevel-explanatory; pedagogical AI competence	AI-Ethics and AI-TCK were leading teacher-level predictors; school digital strategy was an important contextual moderator.	Observational design; causal effects and long-term development not established.	Tier 2, moderate-to-higher; large sample and multilevel modelling, but causal limits remain.
Shi (2025; ref10)	not reported in manuscript	Psychometric instrument development/validation	not reported in manuscript; corpus instrument samples ranged 240–620	Teacher survey responses to generative-AI competence items	EFA/CFA and conventional psychometric validation	T-GAIC; generative-AI competence areas including prompting, output evaluation, and ethics	Psychometric-validation; GenAI competence content	Developed and validated a teacher generative-AI competence instrument.	Country/language/level generalisability not reported in manuscript; cross-cultural replication needed.	Tier 1, higher-to-moderate; validated instrument, generalisability limits.

Author & year	Country/region	Study type	Sample	Data source	AI/ML/LA method used	Competence dimension examined	Role of the method in synthesis	Main findings	Limitations	Quality assessment
Xiao et al. (2025; ref11)	not reported in manuscript	Professional-development feasibility/intervention study	not reported in manuscript	Hybrid-flexible AI competence training data	Empirical feasibility study of hybrid-flexible training	Teachers' AI competence	PD roadmap; institutional barrier evidence	Hybrid-flexible delivery may address institutional barriers through flexible training options.	Pedagogical and ethical concerns not solved by delivery format; sample/follow-up not reported in manuscript.	Tier 4, moderate-to-lower; feasibility evidence, limited causal/longitudinal certainty.
Zhang et al. (2025; ref12)	not reported in manuscript	Psychometric scale development/validation	not reported in manuscript; 30-item scale	Teacher responses to AI-SRLS items	Psychometric validation grounded in Zimmerman's self-regulation theory	AI self-regulated learning (AI-SRLS)	Psychometric-validation; AI-mediated self-regulated development	Validated a 30-item AI-SRLS scale conceptualising teachers' AI learning self-regulation.	Cross-context and longitudinal validity not reported in manuscript.	Tier 1, higher-to-moderate; strong construct focus, replication needed.
An et al. (2025; ref13)	not reported in manuscript	Psychometric scale development/validation	not reported in manuscript	AI-TPACK scale responses	AI-TPACK scale development and validation	AI-TPACK for human and AI concurrently as teachers	Psychometric-validation; pedagogical AI and human–AI co-teaching	Distinguished human-teacher and machine-teacher dimensions within AI-TPACK.	Sample, context, and cross-cultural validation details not reported in manuscript.	Tier 1, higher-to-moderate; validated construct but broader validation needed.
Chiu et al. (2025; ref14)	not reported in manuscript	Psychometric scale development/validation	not reported in manuscript	Teacher responses to TAICS scale	EFA/CFA; measurement invariance noted in manuscript	AI competence self-efficacy; AI knowledge, readiness, ethics, assessment	Psychometric-validation; foundational literacy, ethics, readiness	Validated TAICS and included responsible AI and self-efficacy dimensions; measurement invariance cited as an example.	Generalisation beyond validation context remains limited.	Tier 1, higher; validation plus invariance evidence reported in manuscript.
Delcker et al. (2025; ref15)	not reported in manuscript	Instrument development/validation	not reported in manuscript	Teacher self-perception instrument data	Evidence-based instrument development; psychometric assessment	Teachers' self-perceptions of AI competence	Psychometric-validation; self-efficacy/readiness/ethics/self-regulation	Developed an instrument for teachers' self-perceived AI competence.	Self-report design and context-specific validation limit transferability.	Tier 1, moderate-to-higher; instrument evidence, self-report limits.
Elizondo-Garcia (2026; ref16)	not reported in manuscript	Scoping review	not reported in manuscript	Medical education and AI/vibe-coding literature	Scoping review	Vibe-coding / AI-assisted coding as teaching competence	GenAI-specific competence content	Identified vibe-coding as an emerging AI-related teaching competence in medical education.	Narrow domain; empirical measurement evidence not reported in manuscript.	Tier 3, moderate-to-lower; useful concept mapping, limited empirical strength.
Ding et al. (2024; ref17)	not reported in manuscript	Professional-development intervention/case-based study	not reported in manuscript	Teacher PD using different case types	Case-based PD design	Teacher AI literacy and AI integration	PD roadmap; foundational AI literacy	Case-based learning supported AI literacy and integration in teacher PD.	Sample, control condition, transfer, and follow-up not reported in manuscript.	Tier 4, moderate; feasible PD evidence but causal strength limited.
Tan et al. (2025; ref18)	not reported in manuscript	Professional-development intervention study	not reported in manuscript	Intelligent-TPACK PD intervention data	PD intervention based on i-TPACK framework	Teachers' AI competence; i-TPACK; ethics in practice	PD roadmap; pedagogical AI competence; ethics-as-practice	Teachers understood algorithmic bias conceptually but struggled to identify ethical issues in classroom application; used as longitudinal/professional learning example.	Control/randomisation, transfer, and long-term robustness not fully reported in manuscript.	Tier 4, moderate; intervention evidence but limited causal certainty.
Ramazanoglu and Akin (2025; ref19)	not reported in manuscript	Psychometric scale development/validation	not reported in manuscript	Teacher responses to AI readiness items	Scale development and validation	AI readiness; self-efficacy/readiness	Psychometric-validation; diagnosis phase of PD roadmap	Developed and validated an AI Readiness Scale for teachers.	Generalisability across country/language/level not reported in manuscript.	Tier 1, higher-to-moderate; validated scale, replication needed.
Brandão et al. (2024; ref20)	not reported in manuscript	Integrative literature review	not reported in manuscript	Teacher PD and generative-AI literature	Integrative literature review	Generative AI professional development; ethics education	PD roadmap; ethics-as-practice; GenAI specificity	Argued ethics should be considered for effective AI education and future-oriented teacher PD.	Empirical effectiveness and longitudinal evidence not reported in manuscript.	Tier 3, moderate; integrative review, synthesis-dependent.
Tan et al. (2025; ref21)	Higher education context; country not reported in manuscript	Quantitative mediation/SEM study	not reported in manuscript	Teachers' AI competence, TPACK, and teaching-performance data	Mediation analysis / SEM	AI competence, TPACK, teaching performance	Pedagogical AI competence; PD roadmap	Investigated TPACK as mediator between teacher AI competence and teaching performance in higher education.	Observational mediation limits causal inference; sample/context details not reported in manuscript.	Tier 1, moderate; SEM-type evidence but causal limits.
Zambrano-Romero et al. (2025; ref22)	International review corpus; not reported in manuscript	Systematic review	Teaching-quality ML studies from 2014–2024; count not reported in manuscript	ML in university teaching-quality literature	Systematic review of ML techniques	Teaching quality of university teachers	Predictive-evaluative method map; limitations/gaps	Identified decision tree, SVM, neural network, ensemble learning, and clustering as common teaching-quality assessment techniques.	Corpus-level review; explainability, fairness, and construct validity gaps remained.	Tier 3, moderate; review supports field-level method mapping.

Author & year	Country/region	Study type	Sample	Data source	AI/ML/LA method used	Competence dimension examined	Role of the method in synthesis	Main findings	Limitations	Quality assessment
Dogan et al. (2025; ref23)	International review corpus; not reported in manuscript	Systematic review	not reported in manuscript	AI professional-development literature	Systematic review of TPACK, designs, and effects	AI PD, TPACK, teacher learning	Evidence-base gap; PD roadmap	Showed self-report and quasi-experimental designs were common and long-term evidence was limited.	Longitudinal and strong causal evidence limited.	Tier 3, moderate; review evidence for design gaps.
Fakhar et al. (2024; ref24)	not reported in manuscript	Systematic review / framework paper	not reported in manuscript	Online continuous professional-development literature	Systematic review toward AI-based CPD framework	AI-based online continuous professional development	PD framework; regional balance/gap evidence	Proposed an AI-based framework for online continuous teacher PD.	Empirical validation of framework not reported in manuscript.	Tier 3, moderate-to-lower; framework-oriented review.
Aravantinos et al. (2026; ref25)	K-12 education; region not reported in manuscript	Systematic review	not reported in manuscript	K-12 AI integration and teacher PD-needs literature	Systematic review	AI literacy and professional-development needs	PD needs; foundational AI literacy; adoption barriers	Identified PD needs for AI integration and indicated technical training alone is insufficient.	Contextual transfer and intervention effectiveness not reported in manuscript.	Tier 3, moderate; review evidence for needs/gaps.
Salas-Pilco et al. (2022; ref26)	International review corpus; not reported in manuscript	Systematic review	30 studies	AI and LA in teacher education literature	Systematic review	AI and learning analytics in teacher education	Descriptive-analytic; LA dashboards and analytics; evidence-base gaps	Identified LA dashboards, behavioural analytics, and digital-literacy diagnostics; reported lack of longitudinal studies.	Review corpus limited to included studies; no new empirical validation.	Tier 3, moderate; transparent study count reported in manuscript.
Lameras and Arnab (2022; ref27)	not reported in manuscript	Exploratory review	not reported in manuscript	AI in education literature	Exploratory review	AI affordances for teachers and professional agency	Descriptive-analytic; professional-learning support; AI with teachers	Mapped affordances of AI to teachers and teacher agency.	Exploratory design; systematic corpus and appraisal details not reported in manuscript.	Tier 3, lower-to-moderate; exploratory mapping.
Yau et al. (2025; ref28)	not reported in manuscript	Learning-analytics / qualitative-network study	In-service teachers; number not reported in manuscript	Interview transcripts from teachers undergoing PD	Epistemic Network Analysis (ENA)	Competence to teach AI; TPACK components	Descriptive-analytic; competence-pattern mapping	Mapped interconnections among TPACK components and quantified unlabelled competence data in LA form.	Sample/context and transferability not reported in manuscript.	Tier 2, moderate; transparent analytic role, limited generalis-ability details.
Ahmad et al. (2022; ref29)	not reported in manuscript	Empirical/descriptive AI-in-education study	not reported in manuscript	Academic and administrative AI role data; source not reported in manuscript	not reported in manuscript	Academic/administrative AI roles; assessment and support practices	AI-integrated professional support; assessment/co-teaching context; adoption barriers	Used to show AI can assist teachers in administrative and academic duties.	Design, sample, method, and quality details not reported in manuscript.	Not fully assessable from manuscript; provisionally Tier 5/contextual evidence.
Simut et al. (2024; ref30)	not reported in manuscript	Structural equation modelling study	not reported in manuscript	Teacher competence modelling data; source not reported in manuscript	SEM	Teachers' competence; ethics/responsible AI use	Psychometric/explanatory; ethics-as-practice	Modelled teacher competence and contributed evidence on ethical/responsible AI competence.	Sample, context, and external validation not reported in manuscript.	Tier 1, moderate; SEM evidence with reporting limits.
Alwakid et al. (2025; ref31)	Outcome-based education context; region not reported in manuscript	Empirical AI-and-competence study	not reported in manuscript	Instructional planning and student-performance data; source not reported in manuscript	not reported in manuscript	Teacher competence in AI-supported instructional planning	AI-integrated PD/intervention evidence; assessment and human–AI co-teaching	Explored AI and teacher competence in instructional planning and student performance.	Design, sample, method, and follow-up details not reported in manuscript.	Not fully assessable from manuscript; provisionally Tier 4/5 lower-to-moderate.
Xu et al. (2025; ref32)	TCSL / second-language teacher education; region not reported in manuscript	Framework/instrument-oriented empirical study	Preservice TCSL teachers; number not reported in manuscript	Technology-integration competence data; source not reported in manuscript	Content-based AI-inclusive framework / TPACK-derived analysis	Technology integration competence; AI-inclusive TPACK	Pedagogical AI competence; competence synthesis map	Investigated pre-service TCSL teachers' technology integration competence through an AI-inclusive framework.	Sample and cross-context validation not reported in manuscript.	Tier 1/3, moderate; framework/construct evidence, reporting limits.
Lee and Bryan (2025; ref33)	Teacher education context; region not reported in manuscript	Teacher-education / PD intervention study	Preservice teachers; number not reported in manuscript	AI integration in teacher education data; source not reported in manuscript	not reported in manuscript	Preservice teacher competence; AI literacy/integration	PD roadmap; foundational AI literacy and GenAI specificity	Explored the impact of integrating AI in teacher education on preservice teacher competence.	Design, comparison, transfer, and follow-up details not reported in manuscript.	Tier 4, moderate-to-lower; PD evidence with reporting limits.

Supplementary Table S2. Mapping of Results Categories to Extraction-Matrix Rows

The synthesis categories in the Results and Proposed Synthesis sections were generated by grouping the study-level codes in Supplementary Table S1. This table shows how each category maps back to the row identifiers in the extraction matrix.

Table 2: Mapping between synthesis categories and extraction-matrix rows.

**Synthesis category**	**Extraction-matrix rows**	**How the rows ground the category**
Predictive-evaluative AI/ML role	ref4, ref5, ref7, ref8, ref22	These rows use or review ML models for teacher academic development, teaching-quality classification, neural-network interaction analysis, or competence modelling.
Multilevel-explanatory AI/ML role	ref6, ref9	These rows use regularised or multilevel modelling to explain predictors of PD perception or AI-TPACK development.
Descriptive-analytic LA/review role	ref26, ref27, ref28	These rows map AI/LA patterns, dashboards, affordances, or epistemic networks rather than ranking teachers.
Psychometric-validation role	ref10, ref12, ref13, ref14, ref15, ref19, ref21, ref30, ref32	These rows operationalise competence constructs through scale development, EFA/CFA, SEM, mediation, or framework-based measurement.
Professional-development/support role	ref3, ref11, ref17, ref18, ref20, ref23, ref24, ref25, ref31, ref33	These rows inform the PD roadmap and show how AI competence is developed or supported in teacher learning contexts.
Foundational AI literacy stratum	ref10, ref14, ref15, ref16, ref17, ref20, ref25, ref27, ref33	These rows include AI knowledge, AI literacy, generative-AI understanding, case-based AI learning, or teacher-education AI integration.
Pedagogical AI competence stratum	ref9, ref13, ref18, ref21, ref31, ref32	These rows address AI-TPACK, i-TPACK, AI-supported instructional planning, teaching performance, or AI-inclusive technology integration.
Ethical, critical, reflective AI competence stratum	ref14, ref15, ref18, ref19, ref20, ref30	These rows include responsible AI, ethics, bias recognition, readiness, reflective competence, or modelling of teacher competence.
AI-mediated self-regulated development stratum	ref6, ref12, ref15, ref17, ref18	These rows concern PD predictors, AI-SRLS, self-perception, case-based learning, and longitudinal/professional learning processes.
Assessment and human–AI co-teaching stratum	ref1, ref2, ref13, ref14, ref27, ref29, ref31	These rows ground the assessment, automated feedback, administrative support, and human/machine teacher dimensions of the synthesis map.
Research-gap roadmap	ref4, ref5, ref7, ref8, ref10, ref12, ref13, ref14, ref15, ref18, ref19, ref22, ref23, ref26, ref30, ref32	These rows identify recurring limitations involving construct validity, cross-cultural validation, explainability, fairness, longitudinal evidence, and intervention strength.

Supplementary Table S3. Completed PRISMA-ScR Checklist

This checklist summarises where the PRISMA-ScR reporting items are addressed in the manuscript and supplementary material. Items that are not applicable or not available are marked explicitly.

Table 3: Completed PRISMA-ScR checklist for the scoping review.

**Item**	**Section/topic**	**Where addressed in this manuscript**	**Status**
1	Title	Title identifies the manuscript as a scoping review of artificial intelligence and teacher competence.	Reported
2	Structured summary	Abstract reports the review purpose, evidence source, corpus size, synthesis focus, methods observed, and key limitations.	Reported
3	Rationale	Introduction explains the conceptual fragmentation around AI about teachers and AI with teachers.	Reported
4	Objectives	Introduction lists three scoping questions (RQ1–RQ3).	Reported
5	Protocol and registration	Materials and Methods states that no protocol was prospectively registered.	Not registered
6	Eligibility criteria	Section 2.4 states inclusion and exclusion criteria for teacher-focused AI, ML, and LA competence studies.	Reported
7	Information sources	Section 2.3 identifies Web of Science Core Collection as the sole information source and gives the search date.	Reported
8	Search	Section 2.3 provides full Web of Science field tags, search syntax, limits, language, document type, and publication years.	Reported
9	Selection of sources of evidence	Section 2.4 describes two-stage screening by four reviewers, independent paired screening, disagreement resolution, and N.B. adjudication.	Reported
10	Data charting process	Section 2.5 describes the standardized charting matrix, reviewer charting, second-reviewer checking, consensus discussion, and N.B. consultation.	Reported
11	Data items	Section 2.5 and Supplementary Table S1 list bibliographic, design, participant, region, method, competence, validation, finding, limitation, and quality fields.	Reported
12	Critical appraisal	Section 2.6 states that quality appraisal was optional, conducted after inclusion, and used only to inform interpretation.	Reported
13	Synthesis of results	Section 2.5 describes descriptive mapping, narrative analysis, thematic analysis, comparative analysis, and the two organizing axes.	Reported
14	Selection of sources of evidence: results	Section 2.4 and Figure 1 report 81 records, 39 full texts, and 33 included studies.	Reported
15	Characteristics of sources of evidence	Results and Supplementary Table S1 describe study design, context, sample, data source, method, competence focus, limitations, and quality notes.	Reported
16	Critical appraisal within sources	Section 2.6 and Table 2 present the evidential-strength appraisal used to weight interpretation.	Reported
17	Results of individual sources	Table 1 and Supplementary Table S1 summarise study-level methods, competence focus, main findings, limitations, and synthesis role.	Reported
18	Synthesis of results	Results, Proposed Synthesis, and Supplementary Table S2 report method roles, competence strata, and category-to-row grounding.	Reported
19	Summary of evidence	Discussion and Conclusions summarise the mapped evidence, methodological base, and strength of claims.	Reported
20	Limitations	Methods, Future Research Directions, and Conclusions report database restriction, missing protocol registration, missing screening reliability statistic, limited regional balance, and evidence limitations.	Reported
21	Conclusions	Conclusions state the main implications and caution that outputs are maps of the reviewed corpus rather than evidence of effectiveness across settings.	Reported
22	Funding	Funding statement identifies support from the National Testing Center.	Reported
